# Is Skull Vibration-Induced Nystagmus Useful in Vestibular Neuritis Follow Up?

**DOI:** 10.3390/audiolres12020015

**Published:** 2022-02-26

**Authors:** Ma Piedad García Díaz, Lidia Torres-García, Enrique García Zamora, Ana Belén Castilla Jiménez, Vanesa Pérez Guillén

**Affiliations:** 1 Department of Otorhinolaryngology, Hospital Universitario y Politécnico La Fe, 46026 València, Spain; lidiatorresgarcia@gmail.com (L.T.-G.); ancaji08@gmail.com (A.B.C.J.); vanesaprez2005@yahoo.es (V.P.G.); 2 Department of Otorhinolaryngology, Hospital Vega Baja, 03314 Alicante, Spain; enrique.ga.zam@gmail.com

**Keywords:** vestibular, unilateral vestibular loss, vestibular neuritis, saccades, video head impulse test, vestibular compensation, skull vibration-induced-nystagmus, vestibulo ocular reflex

## Abstract

The aim of this study was to evaluate the vestibulo-ocular reflex (VOR) gain and the saccade regrouping pattern PR score of the Video Head Impulse Test (vHIT) and its relationship with the slow-phase velocity (SPV) of skull vibration-induced nystagmus (SVIN) in recovery after a unilateral vestibular loss (UVL). A total of 36 patients suffering from vestibular neuritis (VN) were recruited and followed up for twelve months. In every visit, horizontal vHIT and an SVIN were performed, as well as VOR gain; PR score and the SPV of SVIN were measured. We observed a positive association between the VOR gain difference and the SPV of SVIN over time (probability greater than 0.86). Additionally, we obtained a positive association between the SPV of SVIN and the PR score in successive visits (odds ratio (OR) = −0.048; CI [0.898, 1.01]), with a probability of 0.95. Our results confirm that SPV of SVIN; VOR gain difference; and PR score decrease over time after a UVL. Both tests are useful in the follow-up of VN, as they could reflect its clinical compensation or partial recovery.

## 1. Introduction

The Video Head Impulse Test (vHIT) and skull vibration-induced nystagmus (SVIN) are two objective tests used to evaluate vestibular function in unilateral vestibular loss (UVL).

The vHIT evaluates the integrity of the vestibulo-ocular reflex (VOR) through two interesting measures: the gain and the saccade regrouping pattern (PR score). The gain reflects the behavior of the VOR according to head movements. The PR score reflects the occurrence and disposition of corrective saccades when VOR gain is low.

Normal VOR gain is close to 1. Patients with UVL have a reduced VOR gain (usually less than 0.8). Impulse tests of their affected ear cause, in these patients, the synchronous movement of their eyes with their head. So, they need to make corrective saccades, thus identifying the side of vestibular loss [1].

In previous studies, it was demonstrated that the time between head movement and the execution of compensatory refixation saccades was related to the degree of vestibular disability. In this work, it is shown that refixation saccades that occur in an organized fashion were associated to lower level of disability and subjective balance [2,3]. The PR score is adapted from the coefficient of variation of the time of appearance of the refixation saccades in all the impulses of the test [3]. The PR score varies between 0 to 100. When refixation saccades appear with a similar time delay in all impulses, the PR value tends towards 0. When refixation saccades appear in a wider time interval after the head impulse, the PR score is close to 100 [3].

Moreover, a 100 Hertz (Hz) bone-conducted vibration applied to either mastoid induces a predominantly horizontal nystagmus with quick-phase beating in the non-affected side in patients with UVL. SVIN is an indicator of vestibular asymmetry and defines the slow-phase velocity (SPV) of the nystagmus and its direction [4]. It is also known that SVIN is related to the severity of vestibular affectation after VN [5,6].

The aim of this study was to evaluate the VOR gain and PR score of the vHIT and its relationship with the SPV of SVIN in the recovery of patients suffering from vestibular neuritis (VN) during the first 12 months after diagnosis.

## 2. Materials and Methods

A prospective longitudinal study was conducted in a tertiary referral center between the years 2014 and 2019. A total of 36 patients suffering from VN were recruited. At least a 12-month follow-up period was required as an inclusion criterion. Patients with spontaneous nystagmus were excluded if the SPV of nystagmus was >2 deg/s.

VN was defined as a vertigo attack for more than 12 h with spontaneous horizontal–torsional nystagmus beating away from the side of the lesion and abnormal HIT and vHIT for the side of the lesion, without hearing or neurological symptoms.

Follow-up was carried out through successive visits after an episode of VN at three, six, and twelve months. In every visit, horizontal vHIT and an SVIN were performed.

vHIT was performed with an Otometrics ICS Impulse equipment (GN Otometrics, Taastrup, Denmark). We stimulated the left or right horizontal semicircular canals with 20 high frequency and short amplitude impulses to measure the VOR gains of both ears. The parameters chosen for the study were the VOR gain difference and PR score. The VOR gain difference was obtained by subtracting the gain of the affected ear from the gain of healthy ear. We have relied on the redacted article by Batuecas et al. to use VOR gain, who supported that this parameter is more likely to be associated with a positive VIN test [5]. The PR score is obtained immediately after testing with the equipment mentioned above for the VOR analysis. We have used only the values of the horizontal semicircular canal to facilitate the comparison of the results in our own study and with the articles published in the literature.

SVIN was evoked by a 100 Hz vibratory stimulus to the affected ear mastoid for 10 s (VVIB 100; Synapsys, Marseille, France). The SPV of the horizontal nystagmus was obtained using videonystagmography software (Ulmer, Marseille, France). SVIN was considered positive when SPV was greater than 2 deg/s and with a frequency of the nystagmus greater than 1 nystagmus/sec [7]. In UVL, SVIN is a good marker of vestibular asymmetry and demonstrates pathological nystagmus beating towards the healthy side in 90% of cases of VN [8].

Statistical analysis was carried out using the R program. First, we analyzed the evolution of different parameters. A Hurdle Gamma regression was assumed to model the excess of zeros (including both the initial zeros and those occurring over time) to study the SVIN’s evolution.

In clinical follow-up, we realized that the recovery from SVIN SPV was not the same in all the patients. At twelve months, there were some patients in whom SVIN SPV became negative, and there were others whose velocity of SVIN SPV decreased but remained positive. In order to elucidate if this difference in SVIN SPV is correlated with differences in VOR gain and/or PR score, we classified patients into two groups according to positivity or negativity of SVIN at 12 months after VN and performed a group analysis.

## 3. Results

Thirty-six patients were included in the study, 23 males and 13 females, with a mean age of 57 ± 11 years. A total of 14 patients suffered a right VN and 22 patients a left VN.

One month after VN, the mean VOR gain difference between both ears was 0.30 ± 0.22, the mean SVIN SPV in the affected ear was 13.12 ± 4.45 deg/s, and the mean PR score was 70.64 ± 26.75. All of them decreased at 12 months, the mean VOR gain difference decreased from 0.30 ± 0.22 to 0.15 ± 0.29, the mean SVIN SPV decreased from 13.12 ± 4.45 deg/s to 1.74 ± 3.41 deg/s in the affected ear, and the mean PR score decreased from 70.64 ± 26.75 to 26.68 ± 22.03.

**Figure 1:**
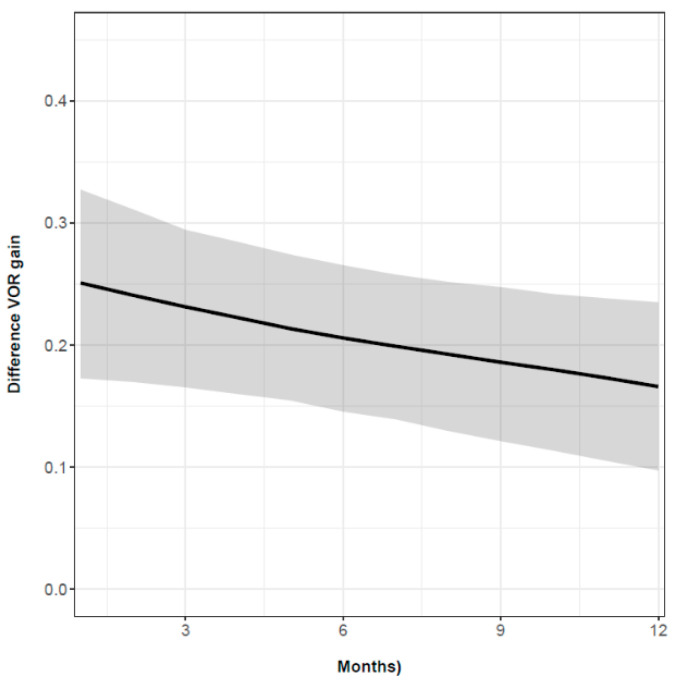
Vestibulo-ocular reflex (VOR) gain difference over time. Vertical axis: VOR gain difference. Horizontal axis: time in months. The line shows the value of the VOR gain difference estimate, and the shaded area indicates the confidence interval (IC).

When we evaluated the vHIT parameters, we found evidence that the VOR gain difference decreased over time (estimate = −0.084, CI 95% [−0.175, −0.001]) (Figure 1). The same result obtained was for PR score, which showed a significant decrease in successive visits (estimate = −1.38, CI 95% [−2.64, −0.17]). Furthermore, we observed that the greater the VOR gain difference, the higher the PR score increased, (*p* < 0.046); thus, there was a positive correlation between the two parameters.

**Figure 2:**
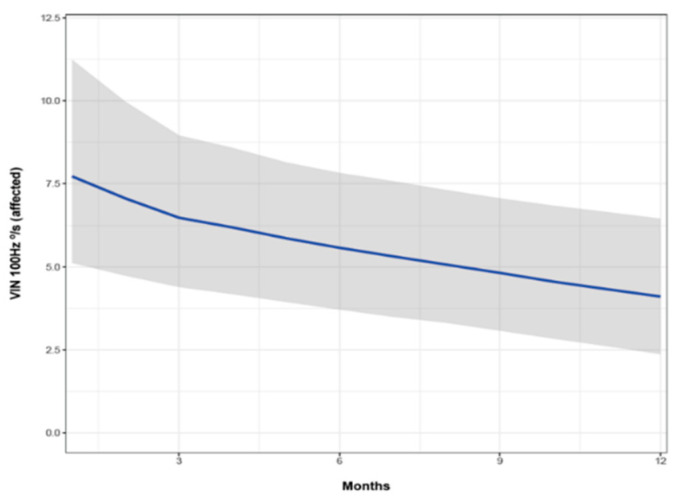
Follow-up of slow phase velocity (SPV) of skull vibration-induced nystagmus (SVIN). Vertical axis: SPV of SVIN at 100 Hz (in the ear affected). Horizontal axis: time in months. The line shows the value of the SVIN SPV estimate, and the shaded area indicate the confidence interval (IC).

All patients included in the study had a positive SVIN in the acute episode of VN. At 12 months, 17 patients were SVIN negative. When we analyzed the SPV of SVIN over time, it tended to become negative, but there was no statistically significant difference (Figure 2). In those patients, SVIN SPV decreased significantly over time (OR = 0.665, CI 95% [0.46, 0.94]).

**Figure 3:**
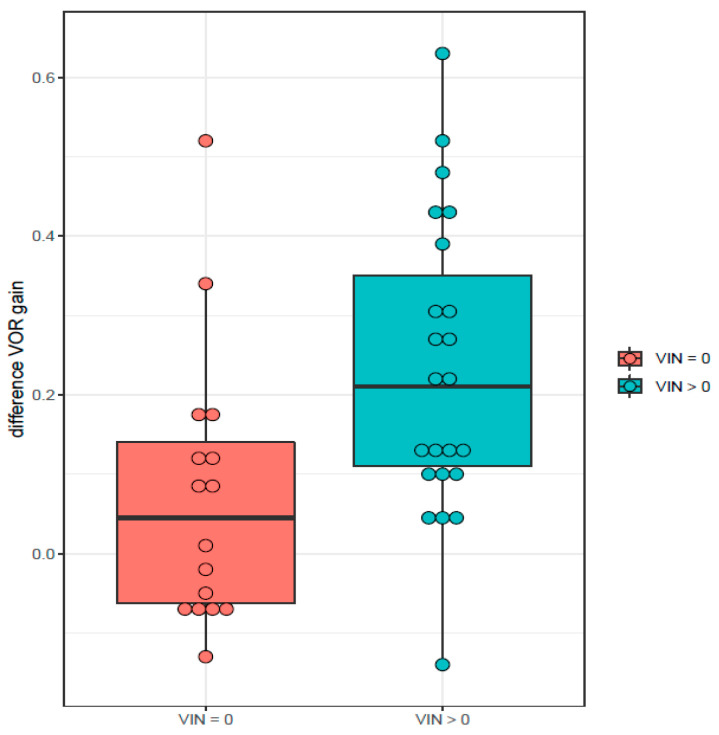
Study groups according to SVIN positivity (VIN > 0) or negativity (VIN = 0) at 12 months after vestibular neuritis (VN) and their VOR gain difference.

**Figure 4:**
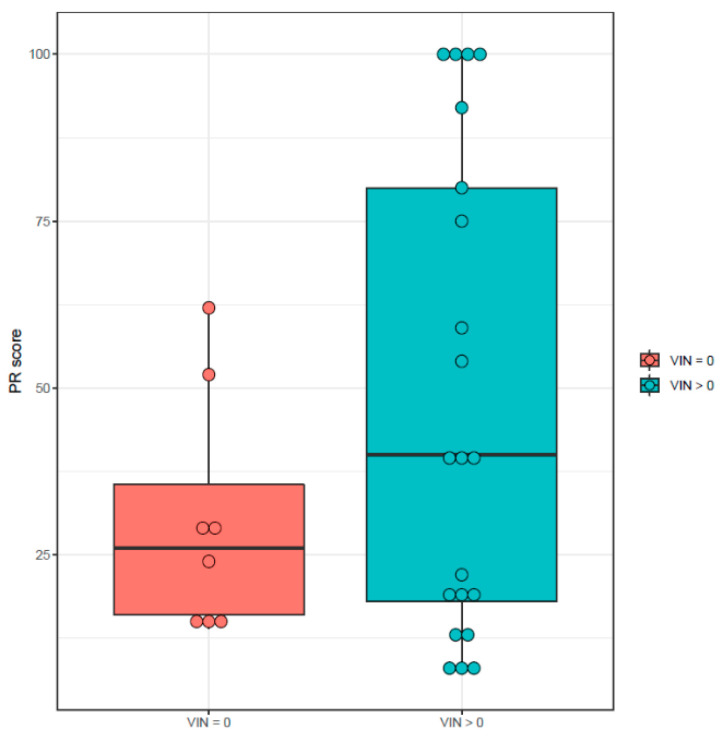
Study groups according to SVIN positivity (VIN > 0) or negativity (VIN = 0) at 12 months after VN and their value of PR score.

Therefore, we classified patients into two groups according to positivity or negativity of SVIN at 12 months after VN. In both groups, we analyzed the VOR gain difference and the PR score. The VOR gain difference was smaller in patients with negative SVIN (estimate = 0.152, CI 95% [0.063, 0.241]) (*p*-value = 0.001) (Figure 3). A similar result was observed in PR score, with values closer to 0 in those patients with negative SVIN (estimate = 6.687, CI 95% [−6.982, 20.357]) (*p*-value = 0.334) (Figure 4). However, the difference between the two groups was not statistically significant.

**Figure 5:**
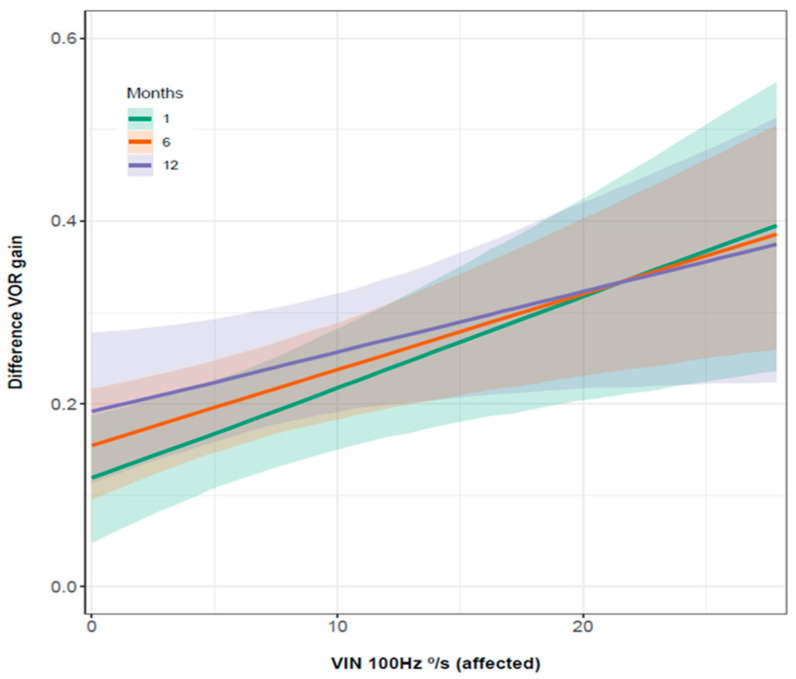
Association between VOR gain difference and SPV of SVIN. Vertical axis: VOR gain difference. Horizontal axis: SPV of SVIN at 100 Hz (in the ear affected). Green line: one month after episode of VN. Orange line: six months after episode of VN. Purple line: twelve months after episode of VN. We can observe that the difference of VOR gain and the SPV of SVIN at 100 Hz decrease as over time.

We observed a positive association between the VOR gain difference and the SPV of SVIN over time (Figure 5) (probability greater than 0.86). Additionally, we obtained a positive association between the SPV of SVIN and the PR score in successive visits (OR = −0.048, CI [0.898, 1.01]), with a probability of 0.95. In successive visits, there was evidence that both values decreased in parallel. In those patients whose gains did not change by more than 0.1 over time, a positive association was also found between SVIN and PR score (OR = 1.009) CI 95% [1.002, 1.015]).

Finally, in patients whose gains were between 0.4–0.59, there was a 0.99 probability of a positive relationship between the PR score and the SPV of SVIN (OR = 1.015, CI 95% [1.003, 1.026]).

## 4. Discussion

The main result of this study was that the SVIN SPV decreases in parallel with the VOR gain difference and the PR score over time, with a statistically significant correlation between them.

Batuecas-Caletrío et al. found that patients with a greater VOR gain difference had a greater SPV of SVIN [9]. We affirm that, in successive visits, these two values evolve in parallel (when the VOR gain difference was greater, the SPV of SVIN was too). Moreover, we observed less VOR gain difference in the negative SVIN’s group. So, we assert that performing vHIT and SVIN together permits us to evaluate objectively the progression of UVL in VN. Recently, some authors confirmed the same observation [9,10].

The aim of this study was focused on the recovery after a VN, and we know that this etiology has a potential recovery. We realized that, in our study, the evolution of SVIN tended to turn negative, results consistent with those found by previous authors [5,11]. Furthermore, in those patients whose test remains positive, their SVIN values decrease over time. Dumas et al. observed in total UVL after surgery or severe chronic compensated UVL patients [10,12] that their SVIN remains stable. This could be explained because they study a permanent vestibular loss.

Matiñó-Soler et al. showed that the PR score decreases over time [3]. We observed that the larger the VOR gain difference, the higher the PR score was. This explains why the refixation pattern of saccades has an effect on vestibular impairment [2]. Indeed, patients who are able to produce isochronous saccadic recovery movements in response to a deficient VOR have less vestibular impairment than those with asynchronous movements [2].

In our study, we aimed to find the relationship between SPV of SVIN and the PR score at successive visits. We observed a positive statistically significant relationship since both values decrease in parallel in successive visits. So, these tests are congruent and homogeneous in their evolution during VN compensation and they are also complementary in the study of vestibular function, as vHIT evaluates the semicircular canals and SVIN is a global vestibular test [10].

We think it is necessary to study the VN evolution in the long term and to relate the results of these objective tests to other subjective tests such the Dizziness Handicap Inventory to consider the clinical status of patients. This would permit us to investigate vestibular compensation. The lack of clinical correlation with supplementary tests (e.g., Caloric test) can be a limitation in this study.

## 5. Conclusions

Our results confirm that the SPV of SVIN, VOR gain difference, and PR score decrease over time after a UVL. In addition, there is a positive association between the SPV of SVIN and VOR gain difference, as well as between the SPV of SVIN and the PR score. Both tests are useful in the follow-up of VN, as they could reflect its clinical compensation or more accurately its partial recovery.

## Data Availability

Not applicable.

## References

[1] Halmagyi G.M., Chen L., MacDougall H.G., Weber K.P., McGarvie L.A., Curthoys I.S. (2017). The video head impulse test. Front. Neurol..

[2] Batuecas-Caletrio A., Santacruz-Ruiz S., Muñoz-Herrera A., Perez-Fernandez N. (2014). The vestibulo-ocular reflex and subjective balance after vestibular schwannoma surgery. Laryngoscope.

[3] E Matiñó-Soler J.R., Pérez Fernández N. (2016). A new method to improve the imbalance in chronic unilateral vestibular loss: The organization of refixation saccades. Acta Otolaryngol..

[4] Dumas G., Perrin P., Schmerber S. (2008). Nystagmus induced by high frequency vibrations of the skull in total unilateral peripheral vestibular lesions. Acta Otolaryngol..

[5] Park H.J., Shin J.E., Shim D.B. (2007). Mechanisms of vibration-induced nystagmus in normal subjects and patients with vestibular neuritis. Audiol. Neurotol..

[6] Hamid M. (2008). Comments on: Clinical significance of vibration-induced nystagmus and head-shaking nystagmus through follow-up examinations in patients with vestibular neuritis. Otol. Neurotol..

[7] Gamarra M.F.V., Krstulovic C., Guillén V.P., Pérez-Garrigues H. (2017). Ipsilesional Nystagmus Induced by Vibration in Subjects with Ménière’s Disease or Vestibular Schwannoma. Otol. Neurotol..

[8] Dumas G., Quatre R., Schmerber S. (2021). How to do and why perform the skull vibration-induced nystagmus test. Eur. Ann. Otorhinolaryngol. Head Neack Dis..

[9] Batuecas-Caletrío A. (2020). Skull vibration-induced nystagmus in vestibular neuritis. Acta Otolaryngol..

[10] Dumas G., Curthoys I.S., Lion A., Perrin P., Schmerber S. (2017). The skull vibration-induced nystagmus test of vestibular function-A. review. Front. Neurol..

[11] Choi K.D., Oh S.Y., Kim H.J., Koo J.W., Cho B.M., Kim J.S. (2007). Recovery of vestibular imbalances after vestibular neuritis. Laryngoscope.

[12] Dumas G., Karkas A., Perrin P., Chahine K., Schmerber S. (2011). High-frequency skull vibration-induced nystagmus test in partial vestibular lesions. Otol. Neurotol..

